# A dyadic approach to understanding anxiety and child overall health in pediatric oncology: insights from an actor–partner interdependence model

**DOI:** 10.1007/s11136-026-04312-x

**Published:** 2026-06-15

**Authors:** Anna L. Olsavsky, Madeline R. Peek, Kathleen E. Montgomery, Micah A. Skeens

**Affiliations:** 1 The Abigail Wexner Research Institute, Nationwide Children’s Hospital, Columbus, OH USA; 2 School of Nursing, University of WI–Madison, Madison, WI USA; 3 College of Medicine, The Ohio State University, 431 South 18Th Street, NEOB 3Rd Fl, Columbus, OH 43205 USA

**Keywords:** Child, Caregivers, Anxiety, Pediatric oncology, Overall health, Actor–partner interdependence model

## Abstract

**Purpose:**

Though child and caregiver anxiety are known to contribute to child overall health, this has not been examined dyadically. This cross-sectional study dyadically examined anxiety (child-self, caregiver-self) and child overall health (child-self, caregiver-proxy) in pediatric cancer.

**Methods:**

Children and caregivers (*n* = 76 dyads; *n* = 152) from two Midwestern children’s hospitals self- or proxy-reported child overall health, and their own anxiety. Descriptive statistics and bivariate analyses (e.g., correlations, t-tests) examined associations among demographics, medical characteristics, and focal variables. An Actor–Partner Interdependence Model (APIM) evaluated dyadic associations between child and caregiver self-reports of anxiety (independent variables) and child self- and caregiver proxy-reports of child overall health (dependent variables). Associations between independent and dependent variables for one reporter are actor effects, whereas across reporters are partner effects.

**Results:**

Among 76 child (88.2% White; 34.2% Female; *M* = 13.35-years-old, *SD* = 3.20) and caregiver (92.1% White, 69.7% Female) dyads, children (*M* = 46.64, *SD* = 9.09) and caregivers (*M* = 39.33, *SD* = 8.97) reported below-average child overall health. Children reported below-average anxiety levels (*M* = 46.31, *SD* = 10.05), and caregivers reported above-average anxiety levels (*M* = 55.18, *SD* = 8.37). APIM analyses revealed significant actor effects: higher levels of self-reported anxiety were associated with lower levels of child overall health (*b* = − 0.29, *p* < .001). Marginally non-significant partner effects revealed greater anxiety was linked to lower partner reports of child overall health (*b* = − 0.14, *p* = 0.051).

**Conclusion:**

Our findings underscore supporting the family system within pediatric cancer and suggest mitigating caregiver anxiety may have downstream benefits. Interventions are needed for caregivers and children to support their mental and overall health during cancer treatment.

## Abstract (plain language summary)

Although previous research shows child and caregiver anxiety contribute to children with cancer’s overall health, no research has examined both child and caregiver reports of anxiety and child overall health at the same time. This limits our understanding of how related these experiences might be across family members, and the ability to provide the right kind of support for families of children with cancer. We found that child and caregiver anxiety are related, and both contribute to child and caregiver perceptions of child overall health. This means that supporting both child and caregiver mental health could ultimately help child overall health during pediatric cancer, but more research is needed to confirm these findings in a larger sample and over time.

## Introduction

Approximately 15,000 children and adolescents are diagnosed with cancer annually in the United States (U.S.) [1]. Cancer treatment advancements have resulted in a 5-year survival rate over 85% [2]. However, pediatric cancer patients face various disease and treatment-related challenges and symptoms, such as fatigue, pain, nausea, and vomiting [8, 9], negatively impacting their overall health and quality of life (QOL) [3–5]. QOL and overall health examine physical, mental, emotional, and social wellbeing and are strongly correlated [6, 7]. Symptoms, stressors due to maintaining daily routines, and disease uncertainty [10] can impair patients’ QOL and worsen anxiety and depressive symptoms [11, 12]. Indeed, anxiety prevalence rates in pediatric cancer patients are 13.92% (range: 4.35–35.14%) [13], and can cause feelings of excessive worry, discomfort, and changes in blood pressure [14]. Additionally, anxiety can negatively impact pediatric cancer patients’ psycho-social wellbeing and QOL [15], as it creates social restrictions, poor academic performance, worse family functioning, and increased risk for other psychological disorders [3]. Furthermore, caregivers report older children with cancer experience more anxiety and cancer-related stress [15], and worse QOL than younger patients [16]. However, all cancer patients are at higher risk of anxiety, but living alone, earlier age at diagnosis, advanced disease, social isolation, and a history of another health condition increases the risk [14].

Pediatric cancer patients’ anxiety levels range widely, which may be explained by the use of different measures, confounding variables, inconsistent reports of risk factors, and biases based on reporter type (i.e., self- versus proxy-report) [9, 15]. Although caregiver proxy-reports offer valuable insights into a child’s QOL and symptoms, they often overestimate symptoms and underestimate abilities relative to child self-reports [9, 17, 18]. Family Systems Theory (FST) postulates individuals influence and are influenced by their family system [19, 20], which may explain these discrepancies; caregivers and children experience cancer as an individual stressor [21], but also as a family system. Therefore, family members’ wellbeing is interconnected. Indeed, multiple models propose that caregiver adjustment and child outcomes are mechanistically linked via shared environment, caregiver–child attachment relationship, and psychobiological processes [22–24]. Therefore, pediatric cancer patients are negatively impacted by poorer caregiver mental health [4, 16], and it is imperative to understand how caregivers’ stressors manifest and impact their own and their child’s wellbeing to optimize the family’s overall health on the individual and systematic level.

Caregivers of children with cancer encounter stressors that impact their psychological wellbeing. Specifically, caregivers find the lack of control and uncertainty for their child’s survival and health stressful [10]. As caregivers are often the main recipient of cancer-related information [25], another common stressor is communicating with their child about cancer, especially for younger children [25, 26]. Additionally, caregivers report financial stress and maintaining daily functioning as common stressors [27]; those of high educational attainment [25], with poor social support, and close to diagnosis report high levels of stress, anxiety, and depression [3, 28–30]. These stressors manifest into internalizing symptoms (i.e., anxiety, depression, post-traumatic stress) in up to 51% of caregivers with a child with cancer [31]. However, caregivers often resist seeking care for themselves, creating a negative impact on their QOL, marriage, mental and physical health, family functioning, and ability to provide social support for up to 5 years post-diagnosis [31–34]. Therefore, as suggested via FST, it is important to consider caregivers’ wellbeing and how it may affect their children and overall family functioning [25].

Recent systematic reviews emphasize the link between caregiver internalizing symptoms and poorer outcomes for children with cancer, including lower QOL and worse social and behavioral adjustment [4, 26, 35, 36]. However, these reviews, and their associated papers, predominantly examine associations between caregiver wellbeing and child overall health using a single reporter (e.g., mother only) rather than modeling the interdependence between caregivers and children; thus, findings could reflect reporter biases. To address these biases, some studies have examined associations between caregiver and child wellbeing dyadically using an Actor–Partner Interdependence Model (APIM) to account for interdependence in family caregiver–child dyads [37]. APIMs are designed to examine dyads, a limitation of standard statistical techniques which assume independence. In an APIM, associations between independent and dependent variables for one reporter are actor effects, whereas across reporters are partner effects. In the literature, APIMs have revealed that more perceived social support is suggested to dyadically improve caregiver and child overall health [32]. Additionally, as children’s uncertainty about their illness increases, caregivers’ QOL decreases; contrarily, caregivers’ uncertainty is unrelated to patients’ QOL [10]—a finding that is inconsistent with other literature that posits caregivers’ psychological wellbeing influences children’s overall health [3, 16, 38]. Overall, APIMs provide a nuanced depiction of factors influencing caregiver and child wellbeing, and previous studies have not dyadically examined these associations between wellbeing and overall health. Therefore, the complex dynamic between caregivers and their children with pediatric cancer requires additional evaluation.

Thus, this study aimed to examine caregiver and child anxiety with caregiver and child perceptions of child overall health among families experiencing pediatric cancer. We hypothesized that greater anxiety would have significant actor and partner effects, contributing to worse child overall health. Additionally, as called upon by Neugebauer and Mastergeorge [26], this study uses an APIM to examine the interdependent associations between children with cancer and their caregivers. While forementioned studies acknowledge the interconnection between caregivers’ psychological wellbeing and their child’s overall health, some utilized caregiver self- and proxy-reports [3, 16, 33], and others utilized self-reports for both the children and caregivers [10, 28, 32]. Therefore, to fully understand child overall health, it is imperative to consider the child within their family system by examining the roles of both child and caregiver anxiety.

## Methods

### Procedures

This paper presents a secondary analysis of children and caregivers recruited between October 2023 and March 2024 from two American, Midwestern, freestanding children’s hospitals. This cross-sectional, minimal risk, pilot study was approved by the University of Wisconsin-Madison Health Sciences Institutional Review Board (IRB#2023-0921). For children, eligibility criteria included: (a) aged 8–17 years, (b) cancer diagnosis, (c) either currently receiving adjuvant cancer treatment for ≥ 2 months or completed treatment ≤ 6 months, and (d) English literate. Caregivers were (a) ≥ 18 years old, (b) caring for a child with cancer with the forementioned eligibility criteria, and (c) English literate. Caregivers who were legal guardians and present during an inpatient chemotherapy admission or outpatient oncology visit were recruited; families were not asked to identify a “primary” caregiver as this inclusion criteria has contributed to exclusion of fathers [39]. Exclusion criteria included conditions preventing children or caregivers from independently completing surveys (i.e., developmental delays), or if the child’s cancer treatment only included radiation or surgery, as the original study aimed to understand symptom experiences from chemotherapy. Informed consent was obtained from caregivers for themselves and their child. Per institutional standards for minimal risk studies, children provided verbal assent. Surveys were completed individually during the visit or remotely within 30 days (+ 3 business days) via Research Electronic Data Capture (REDCap). Children and caregivers were compensated with a $25 gift card ($50/dyad) upon study completion. As a pilot study, sample size was determined based on the award scope.

### Measures

#### Demographic and medical characteristics

Caregivers reported on their own and their child’s demographic characteristics, including child and caregiver age (calculated using birthdates and study dates with retained decimals), child sex, caregiver gender, number of children in household, number of caregivers, caregiver education, and caregiver income. The research team extracted child medical characteristics from electronic medical records, including cancer type, time since diagnosis, relapse status, cancer therapy intensity, disease status within the previous 10 days, and cancer treatment status within the previous 10 days.

#### Child overall health

Children completed the PROMIS Pediatric Scale v1.0 Global Health 7 [6] and caregivers completed the PROMIS Parent Proxy Scale v1.0 Global Health 7 [6] to evaluate child overall health. Seven items were rated on a 1–5 scale and then converted to *T*-scores (child range: 16.0–67.5, caregiver range: 14.7–66.1; higher scores = better overall health). For caregivers and children, 50 is the average U.S. population score. Scores > 50 indicate above-average child overall health. Previous literature confirms acceptable reliability/validity [6, 7], further supported by our sample’s internal consistency (children: α = 0.76; caregivers: α = 0.81). Both caregiver and child questionnaires measured general health, QOL, physical health, mental health, sadness, enjoyment, and being heard (e.g., “How often do you [or your child] have fun with your friends?”).

#### Anxiety

Children self-reported their anxiety using the PROMIS Pediatric Short Form v2.0 Anxiety 8a scale [40, 41] and caregivers self-reported their anxiety using the PROMIS Short Form v1.0 Anxiety 8a scale [40, 41], which included 8 items on a 5-point Likert scale. Items were converted into an overall *T*-score (child range: 33.5–83.3, caregiver range: 37.1–83.1; higher scores = more anxiety). For both caregivers and children, 50 is the average U.S level of anxiety. Previous literature confirms acceptable reliability/validity [40, 41], further supported by our sample’s internal consistency (children: α = 0.90; caregivers: α = 0.92). Both caregiver and child anxiety questionnaires measured variations of fear, nervousness, and worry (e.g., “I felt uneasy…”).

### Statistical analysis

Using IBM SPSS Statistical Software (version 28) [42], we analyzed descriptive statistics to describe sample characteristics and normality of focal variables. This included demographic information (i.e., child and caregiver age, child sex, caregiver gender, number of children in household, number of caregivers, caregiver education, caregiver income), medical characteristics (i.e., cancer type, time since diagnosis, relapse status, disease status within the previous 10 days, cancer treatment status within the previous 10 days, cancer therapy intensity), and mean levels of focal variables (i.e., child and caregiver self-reports of anxiety, child self- and caregiver proxy-reports of child overall health). Skewness was between − 0.5 and + 0.5 for anxiety and child global health, and kurtosis was between − 1 and + 1, indicating the distribution of data was not severely non-normal [43, 44]. We conducted bivariate analyses to understand associations and differences among demographic information, medical characteristics, and focal variables using Pearson, point-biserial, and Spearman’s rank correlations (for variables with skewness or kurtosis less than − 1.0 or greater than 1.0), independent and paired samples *t*-tests, and a one-way ANOVA. Across analyses, *p* < 0.05 was considered statistically significant. To avoid strict dichotomization [45–47] in determining significance and to consider potentially clinically, yet not statistically, significant findings [48, 49], we report marginally non-significant results (*p* = 0.05 to *p* < 0.10). Due to the limited power in these secondary analyses, a lack of significance may not evince null results [50].

Finally, we analyzed an APIM using MPlus version 8.11 to understand dyadic associations between child and caregiver self-reports of anxiety (independent variables) and child self- and caregiver proxy-reports of child overall health (dependent variables) [37]. As caregivers and children were nested within families, they were non-independent [37], though small correlations between caregiver and child reports of focal variables also supported non-independence. Evaluating these data using an APIM allows for the modeling of non-independent data, by simultaneously considering correlations among independent variables and between residual variance of dependent variables [37]. Children and caregivers were treated as theoretically distinguishable on the meaningful factor of being either a caregiver or child, though significant differences between focal variables also supported empirical distinguishability [37]. Actor effects are associations between variables reported on by the same dyad member (e.g., child anxiety and self-reported child overall health), whereas partner effects are associations across dyad members (e.g., caregiver anxiety and child self-reported child overall health). This model included covariates based on significant bivariate analyses with anxiety and/or child overall health (i.e., child age, child relapse status, caregiver education, caregiver gender). First, an unconstrained, saturated model (i.e., fully identified; no fit statistics available) was evaluated [37, 51]. Then, to achieve parsimony, increase statistical power, and allow for evaluating model fit, constraints were systematically tested across like paths [37, 51]. A significant change in chi-square (*p* < 0.05) indicated paths were not equivalent, in which case the constraint was not retained. Good model fit for the constrained model was determined based on a non-significant chi-square (*p* > 0.05), a comparative fit index (CFI) > 0.95, a Tucker-Lewis Index (TLI) > 0.95, a root-mean-square error of approximation (RMSEA) < 0.06, and a standardized root-mean-squared residual (SRMR) < 0.08 [52]. Low levels of missing data (0–7% by variable) were estimated using full information maximum likelihood [53]. Independent variables and covariates, as well as the error terms of dependent variables, were allowed to covary across all models [37, 51].

## Results

### Descriptive statistics

Of 106 eligible dyads, 6 dyads were unapproachable (e.g., no appointment while eligible), 11 dyads declined participation, and 89 consented. Thirteen dyads did not complete the study due to requesting a remote visit and subsequently never completing surveys. Therefore, 76 family dyads consisting of one caregiver and one child with cancer participated (*n* = 152). Caregivers were an average of 42.17 years old (*SD* = 7.44), more female than male, and had completed some education past high school. Children were an average of 13.35 years old (*SD* = 3.20), and 17 (22.4%) had relapsed. Children were diagnosed with hematologic malignancies (*n* = 40, 52.6%), central nervous system (CNS) tumors (*n* = 14, 18.4%), and non-CNS solid tumors (*n* = 22, 28.9%). Table 1 displays full demographic and medical information. Caregivers reported their children had below average overall health (*M* = 39.33, *SD* = 8.97), whereas children reported their overall health as significantly higher than caregivers (*M* = 46.63, *SD* = 9.09; *t*(75) = 5.37, *p* < 0.001), yet both means were below the population average of 50. Caregivers self-reported slightly higher than average anxiety (*M* = 55.18, *SD* = 8.37), and children reported slightly lower than average anxiety (*M* = 46.31, *SD* = 10.05); child anxiety was significantly lower than caregiver anxiety (*t*(75) = 5.98, *p* < 0.001).

**Table 1:** Demographic characteristics

Demographic characteristics	Caregiver (n = 76)	Child (n = 76)
*Gender (caregiver)/sex (child)*		
Male	22 (28.9%)	50 (65.8%)
Female	53 (69.7%)	26 (34.2%)
*Race*		
White	70 (92.1%)	67 (88.2%)
Black or African American	2 (2.6%)	3 (3.9%)
Vietnamese	1 (1.3%)	1 (1.3%)
American Indian or Alaskan Native	–	1 (1.3%)
Other	3 (3.9%)	3 (3.9%)
*Ethnicity*		
Not Hispanic or Latino	66 (86.8%)	66 (86.6%)
Mexican or Chicano	7 (9.2%)	6 (7.9%)
Another Hispanic, Latino, or Spanish Origin	1 (1.3%)	2 (2.6%)
*Relationship to the child*		
Biological caregiver	69 (90.8%)	–
Step-caregiver	2 (2.6%)	–
Adoptive caregiver	3 (3.9%)	–
Grandparent	1 (1.3%)	–
Other caregiver	1 (1.3%)	–
*Caregiver highest grade of school completed*		
Less than high school	4 (5.3%)	–
High school	12 (15.8%)	–
Post high school trade	26 (34.2%)	–
College	16 (21.1%)	–
Graduate school	16 (21.1%)	
*Number of adults providing child-care*		
1 Adult	12 (15.8%)	–
2 Adults	60 (78.9%)	–
3 Adults	3 (3.9%)	–
5 + Adults	1 (1.3%)	–
*Number of children in the house*		
1 Child	6 (7.9%)	–
2 Children	30 (39.5%)	–
3 Children	19 (25.0%)	–
4 Children	10 (13.2%)	–
5 Children	7 (9.2%)	–
6 + Children	4 (5.3%)	–
*Annual family income*		
Under $25,000	5 (6.6%)	–
$25,001—$50,000 per year	9 (11.8%)	–
$50,001—$75,000 per year	15 (19.7%)	–
$75,001 -100,000 per year	10 (13.2%)	–
$100,001—$150,000 per year	13 (17.1%)	–
$150,001 or more	16 (21.1%)	–
Age (mean, SD, range)	43.17, 7.44, 27.4–60.3	13.4, 3.2, 8.1–17.9
Days since diagnosis (Mean, SD, range)	–	431.4, 412.4, 63–1995
*Relapse status*		
Yes	–	17 (22.4%)
No		59 (77.6%)
*Disease status in 10 days prior*		
Yes (presence of any disease)	–	50 (65.8%)
No	–	24 (31.6%)
*Diagnosis type*		
Hematologic malignancy	–	40 (52.6%)
Non-CNS solid tumor	–	22 (28.9%)
CNS tumor	–	14 (18.4%)
*Cancer-directed treatment in 10 days prior*		
Yes	–	46 (60.5%)
No	–	30 (39.5%)
Cancer treatment intensity (Mean, SD, range)	–	1.58, 0.80, 0–3

### Bivariate analyses

Pearson, point-biserial, and Spearman’s rank correlations were used to examine bivariate associations among focal variables and demographic and medical characteristics (Table 2). Better self-reported child overall health was significantly associated with lower child anxiety and younger child age. Better caregiver proxy-reported child overall health was associated with lower caregiver self-reported anxiety and caregiver education. Higher caregiver anxiety was associated with greater caregiver education. Additionally, independent samples t-tests examined differences in child and caregiver reports of anxiety and child overall health based on child sex, caregiver gender, child relapse status, child disease status, and child treatment status (Table 3), revealing children reported lower child overall health with a male caregiver and a trend of female caregivers reporting higher anxiety and lower child overall health. Caregivers reported more anxiety when their child had relapsed disease. Finally, four one-way ANOVAs were examined to determine the effect of diagnosis types (i.e., hematological malignancy, CNS tumor, non-CNS tumor) on focal variables; all four tests revealed no significant differences by diagnosis type (*p*’s = 0.27–0.74). Significant correlates of focal variables were covariates in the APIM: child age, caregiver education, caregiver gender, and child relapse status.

**Table 2:** Pearson and Spearman’s rank correlations between child overall health, anxiety, and continuous key demographic and medical characteristics

	*M*	*SD*	Child self-reported child overall health	Caregiver proxy-reported child overall health	Child self-reported anxiety	Caregiver self-reported anxiety
Child self-reported child overall health	46.63	9.09	–			
Caregiver proxy-reported child overall health	39.33	8.97	0.143	–		
Child self-reported anxiety	46.31	10.05	− 0.309**	− 0.212^†^	–	
Caregiver self-reported anxiety	55.18	8.37	− 0.034	− 0.307**	0.021	–
**Child age**			− 0.371**	− 0.066	0.027	− 0.091
Caregiver age			− 0.198^†^	− 0.008	0.035	− 0.042
**Children in household**			0.083	− 0.027	− 0.099	− 0.031
**Adults caring for child**			0.017	− 0.195	− 0.125	0.130
**Caregiver education**			0.030	− 0.245*	0.010	0.275*
**Caregiver income**			0.082	0.011	− 0.046	0.196^†^
**Child time since diagnosis**			0.111	0.012	− 0.150	− 0.142
**Child cancer treatment intensity**			0.031	− 0.025	− 0.125	− 0.001

^*^Correlation is significant at the *p* < 0.05 level

^**^Correlation is significant at the *p* < 0.01 level

^†^Correlation is marginally non-significant at the *p* < 0.10 level

Bolded variables use a Spearman’s rank non-parametric correlation coefficient due to Skewness or kurtosis values greater than 1.0 or less than − 1.0

**Table 3:** T-tests between child overall health, anxiety, and binary key demographic and medical characteristics

			Child self-reported child overall health					Caregiver proxy-reported child overall health					Child self-reported anxiety					Caregiver self-reported anxiety				
Variable	Group	*N*	*M*	*SD*	*t*	*df*	*p*	*M*	*SD*	*t*	*df*	*p*	*M*	*SD*	*t*	*df*	*p*	*M*	*SD*	*t*	*df*	*p*
Child sex	Male	49	46.58	8.36	0.29	73	0.774	40.21	9.30	− 1.21	73	0.232	4.27	0.94	0.37	73	0.713	55.66	7.83	− 0.54	73	0.589
	Female	26	47.22	10.33				37.58	8.39				4.35	1.08				54.55	9.47			
Caregiver gender	Male	22	44.10	5.54	2.13	66.7	0.037*	42.41	8.97	− 1.96	73	0.053^†^	4.27	0.88	0.13	73	0.896	52.69	8.91	1.74	73	0.086^†^
	Female	53	47.93	9.96				38.00	8.81				4.31	1.03				56.35	8.00			
Child relapse status	None	59	46.26	9.29	− 0.65	74	0.519	39.85	9.28	0.93	74	0.358	4.21	0.95	− 1.22	74	0.225	54.11	8.41	− 2.12	74	0.037*
	Relapsed	17	47.89	8.53				37.56	7.79				4.54	1.08				58.89	7.33			
Child disease status 10 days prior	Absent	24	45.88	9.14	− 0.71	72	0.479	39.66	7.71	0.21	72	0.838	4.14	1.04	− 1.02	72	0.312	54.32	8.56	− 0.57	72	0.568
	Present	50	47.48	8.95				39.19	9.74				4.39	0.96				55.51	8.29			
Child cancer treatment status 10 days prior	Absent	30	44.59	9.59	− 1.59	74	0.116	40.57	7.89	0.97	74	0.337	4.14	0.90	− 1.05	74	0.299	54.80	7.94	− 0.32	74	0.749
	Present	46	47.95	8.60				38.53	9.61				4.38	1.03				55.43	8.72			

^*^Two-sided t-test is significant at the *p* < 0.05 level

^†^Two-sided t-test is marginally non-significant at the *p* < 0.10 level

### APIM results

#### Saturated model

**Fig. 1:**
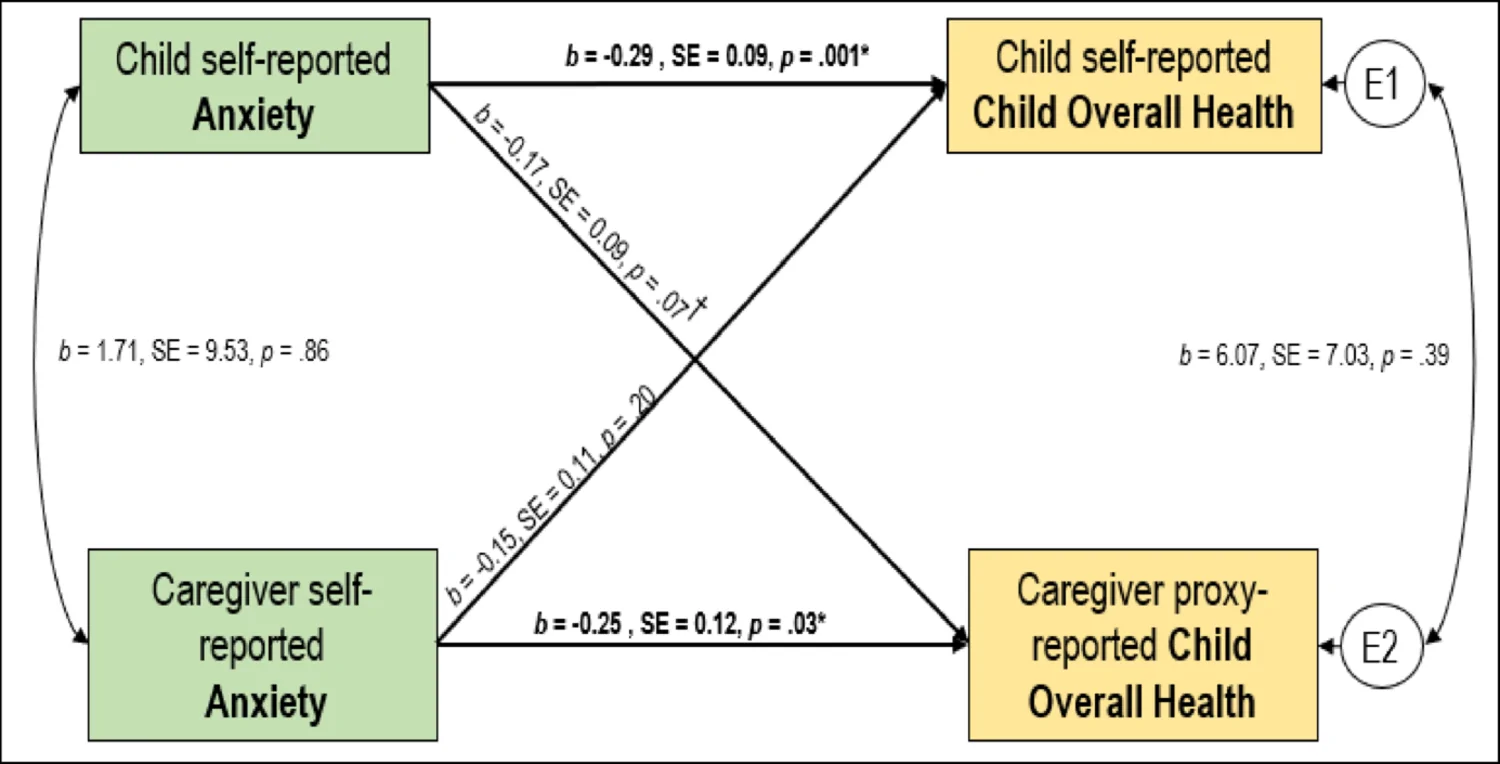
Saturated actor–partner interdependence model of child and caregiver self-reported anxiety and child self- and caregiver proxy-reported child overall health. Values (*b*) represent unstandardized beta coefficients. *SE* is the standard error of the beta coefficient. E1 = residual/error term for child; E2 = residual/error term for caregiver. Independent variables and control variables (not pictured: child age, child relapse status, caregiver gender, and caregiver education) were allowed to covary. The error terms of dependent variables were also allowed to covary. Significant path estimates (*p* < 0.05) have an * and are bolded. Marginally non-significant paths (*p* = 0.05–*p* < 0.10) have an ^†^. Actor–Partner Interdependence Models were analyzed using MPlus version 8.11. Full covariate results can be found in Table 4

The initial, unconstrained, saturated model, for which there are not model fit statistics, revealed significant actor paths from anxiety to child overall health for both children and caregivers. However, partner effects were non-significant, with a marginally non-significant association from child self-reported anxiety to caregiver proxy-reported child overall health and a non-significant association from caregiver self-reported anxiety to child self-reported child overall health. This model included covariates of child age, child relapse status, caregiver gender, and caregiver education. Greater child age and caregiver male gender were associated with worse self-reported child overall health, though no other covariates were significantly associated with self-reported child overall health, and no covariates were associated with caregiver-reported child overall health. Additionally, greater caregiver education and having a child who had relapsed were associated with higher caregiver self-reported anxiety. No other covariates were associated with caregiver or child anxiety (Fig. 1; Table 4).

**Table 4:** Actor–partner interdependence model results

	Saturated model			Constrained model		
	*b*	*SE*	*p*	*b*	*SE*	*p*
*Child self-reported overall health*						
Child self-reported anxiety	− 0.29	0.09	0.001*	− 0.29	0.07	< 0.001*
Caregiver self-reported anxiety	− 0.15	0.11	0.20	− 0.14	0.07	0.05^†^
Child age	− 1.05	0.28	< 0.001*	− 1.05	0.28	< 0.001*
Child relapse status	1.75	2.17	0.42	0.50	1.61	0.76
Caregiver gender	− 4.08	2.08	0.049*	− 4.69	2.03	0.02*
Caregiver education	0.30	0.82	0.71	− 0.35	0.62	0.57
*Caregiver proxy-reported overall health*						
Child self-reported anxiety	− 0.17	0.09	0.07^†^	− 0.14	0.07	0.05^†^
Caregiver self-reported anxiety	− 0.25	0.12	0.03*	− 0.29	0.07	< 0.001*
Child age	− 0.30	0.30	0.31	− 0.30	0.30	0.31
Child relapse status	− 1.13	2.30	0.62	0.50	1.61	0.76
Caregiver gender	2.22	2.18	0.31	2.89	2.13	0.17
Caregiver education	− 1.16	0.87	0.19	− 0.35	0.62	0.57
*Child self-reported anxiety*						
Caregiver self-reported anxiety	1.71	9.53	0.86	1.71	9.53	0.86
Child age	1.06	3.65	0.77	1.06	3.65	0.77
Child relapse status	− 0.38	0.48	0.43	− 0.38	0.48	0.43
Caregiver gender	− 0.60	0.53	0.26	− 0.60	0.53	0.26
Caregiver education	0.19	1.32	0.89	0.20	1.32	0.88
*Caregiver self-reported anxiety*						
Child age	− 2.48	3.05	0.42	− 2.48	3.05	0.42
Child relapse status	0.83	0.41	0.04*	0.83	0.41	0.04*
Caregiver gender	− 0.76	0.44	0.09^†^	− 0.76	0.44	0.09^†^
Caregiver education	2.61	1.14	0.02*	2.62	1.14	0.02*

^*^ *p* < 0.05

^†^* p = 0.05 to p* < 0.10

#### Constrained model

Constraints were evaluated in this order: (1) actor paths from anxiety to child overall health, (2) partner paths from anxiety to child overall health, (3) caregiver education to child self- and caregiver proxy-reports of child overall health, (4) caregiver gender to child self- and caregiver proxy-reports of child overall health, (5) child age to child self- and caregiver proxy-reports of child overall health, and (6) child relapse status to child self- and caregiver proxy-reports of child overall health. Constraints 1, 2, 3, and 6 were retained, whereas constraints 4 and 5 significantly worsened model fit (i.e., there was a significant change in chi-square [*p* < 0.05]) and were not retained; this meant caregiver gender and child age variables had significantly different associations with child overall health by reporter (Table 5). Thus, the constrained model constrained actor and partner paths from anxiety to child overall health, as well as caregiver education and child relapse status to child and caregiver reports of child overall health.

**Table 5:** Actor–partner interdependence model constraint testing

	Change in chi-square	*p*	Retained?
(1) Actor paths from anxiety to child overall health	0.042	0.84	Yes
(2) Partner paths from anxiety to child overall health	0.022	0.88	Yes
(3) Caregiver education to child self- and caregiver proxy-reports of child overall health	1.644	0.20	Yes
(4) Caregiver gender to child self- and caregiver proxy-reports of child overall health	7.063	0.01	No
(5) Child age to child self- and caregiver proxy-reports of child overall health	3.93	0.047	No
(6) Child relapse status to child self- and caregiver proxy-reports of child overall health	0.79	0.37	Yes

**Fig. 2:**
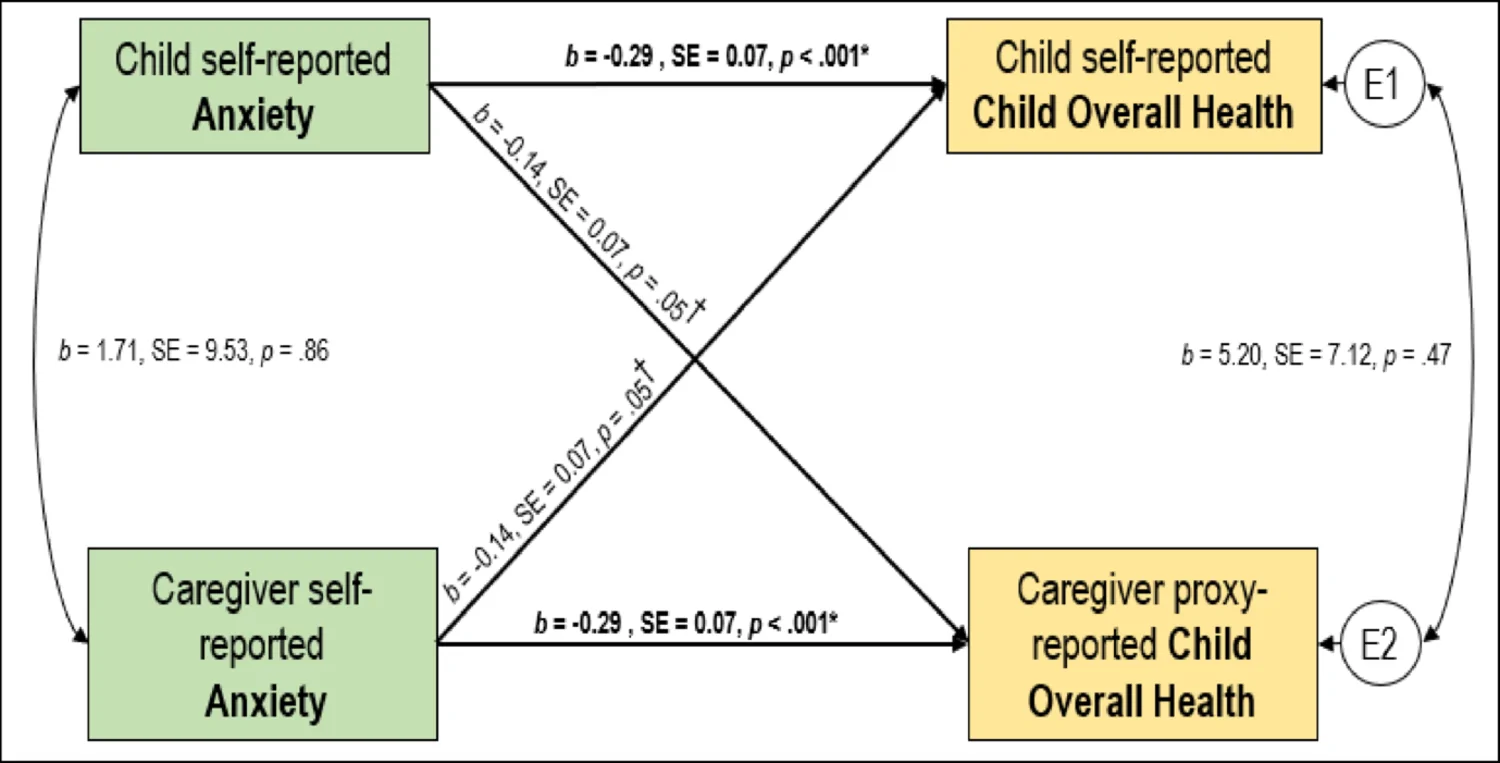
Constrained actor–partner interdependence model of child and caregiver self-reported anxiety and child self- and caregiver proxy-reported child overall health. Values (*b*) represent unstandardized beta coefficients. *SE* is the standard error of the beta coefficient. E1 = residual/error term for child; E2 = residual/error term for caregiver. Independent variables and control variables (not pictured: child age, child relapse status, caregiver gender, and caregiver education) were allowed to covary. The error terms of dependent variables were also allowed to covary. Significant path estimates (*p* < 0.05) have an * and are bolded. Marginally non-significant paths (*p* = 0.05–*p* < 0.10) have an ^†^. Actor–Partner Interdependence Models were analyzed using MPlus version 8.11. Upon constraint testing, two constraints significantly worsened model fit (i.e., there was a significant change in chi-square [*p* < 0.05]) and were not retained: caregiver gender and child age. Full covariate results can be found in Table 4

The final, constrained model indicated good fit, *χ*^*2*^(*df* = 4, *p* = 0.64) = 2.50, RMSEA = 0.00, RMSEA 90% CI [0.00, 0.14], CFI = 1.00, TLI = 1.00, SRMR = 0.02. There was a significant, equivalent actor effect from anxiety to child overall health: as self-reported anxiety increased, child overall health decreased. There was also a marginally non-significant, equivalent partner effect from self-reported anxiety to partner-reported child overall health; greater self-reported anxiety was marginally associated with lower partner-reported child overall health. Male caregiver gender and older child age were significant predictors of lower self-reported child overall health. Greater caregiver education and having a child who had relapsed were significantly associated with higher caregiver self-reported anxiety, and female caregivers had higher anxiety than male caregivers, though this was marginally non-significant. Finally, male caregivers had lower education than female caregivers (Fig. 2; Table 4).

## Discussion

Guided by FST, we investigated interdependent associations between caregiver and child anxiety, and caregiver and child perceptions of child overall health in families experiencing pediatric cancer. Findings indicated that (1) children self-reported higher child overall health than their caregivers, (2) individual anxiety is a significant predictor of one’s perceptions of the child’s overall health, and (3) to a lesser extent, one’s own anxiety may impact the other dyad member’s views of the child’s health. Although previous research has identified links between child anxiety and QOL [15, 16] and recent work has identified caregiver distress as a significant predictor of proxy-symptom reporting [33], these findings have not examined associations between children and caregivers dyadically, limiting the ability to foster tailored, family-centered interventions. Our results suggest mitigating caregiver anxiety could alter their proxy-reports of child overall health and be relevant for children’s perceptions of their overall health.

We found higher levels of caregiver anxiety were associated with caregivers’ perceptions of child overall health and may contribute to their child’s perceptions of their overall health. Though previous research has linked parental distress to child distress and QOL [30, 54], none of these studies utilized both caregiver and child reports simultaneously [4, 26, 35, 36], thereby not accounting for dyadic interdependencies among caregivers and children. Our results support both intrapersonal and interpersonal dynamics in the association between anxiety and child overall health; thus, study designs may benefit from the inclusion of both caregiver and child perspectives, especially considering significant discrepancies in caregiver and child reports of child overall health. Moreover, findings add to a relatively small literature base identifying factors predicting proxy-reports for children [33], and further underscore the importance of collecting patient-reported outcomes in pediatrics [9, 17].

Our focal APIM results revealed higher levels of caregiver anxiety for female caregivers, caregivers of children with relapsed cancer, and caregivers with more education; these associations may offer directions for tailoring caregiver support during pediatric cancer. First, although caregiver anxiety was related to child overall health regardless of caregiver gender, anxiety was lower amongst male caregivers. This finding aligns with known higher levels of anxiety in women than in men [55] and the greater perceived or actual involvement and knowledge of female caregivers in the child’s treatment, contributing to potentially more concerns and associated anxiety [56]. These findings suggest anxiety-mitigating interventions for caregivers and children may not be as beneficial for fathers as they would be for mothers. Perhaps unsurprisingly, caregivers of children with relapsed disease had heightened anxiety, suggesting these caregivers may need additional psychological support compared to those at initial diagnosis; this result could also suggest a need for ongoing caregiver support further from diagnosis [30]. Finally, caregivers with more education also had higher anxiety, which aligns with previous research stating caregivers with higher education have higher distress [25]. However, this could potentially be due to fathers in this sample having both lower anxiety and lower education. Thus, additional research is needed among fathers to better understand and discern the best ways to support caregivers’ mental health based on educational status.

Along with higher levels of child and caregiver anxiety, children reported lower overall health when they were older and had a male caregiver. Previous literature supports that children have lower QOL and overall health when they are older at diagnosis [3, 15, 16]. However, child age was not associated with caregivers’ proxy-report of child overall health; given autonomy contributes to adolescent wellbeing [57], it may be that adolescents are either less likely to express their overall health to caregivers or their autonomy is challenged due to reliance on caregivers during treatment, thereby negatively impacting reports of child overall health. This further supports soliciting patient-reported outcomes, particularly for adolescent patients; older children may need more direct support for their overall health, with younger children perhaps benefiting more from interventions targeting their caregivers [16]. Children with a male caregiver participating also reported lower overall health. Given relatively less research has included fathers, previous literature is scant to help explain this finding [35, 58]. One theory could be fathers become more involved, and therefore more likely to participate in research, when a child has more advanced disease [58]. Ultimately, additional research is needed among fathers of children with cancer to understand the ways by which they influence their child’s perceptions of child overall health.

### Study limitations

This study is not without limitations. Firstly, the sample was modestly-sized, primarily White and middle-to-upper socioeconomic status, and recruited caregivers based on presence at study approach, limiting generalizability. Future research should attempt to recruit more representative samples. Post-hoc power analyses revealed our sample was slightly underpowered (74%) to detect identified actor effects, and very underpowered to detect identified partner effects (24%). Thus, future research should aim for larger sample sizes to obtain adequate power and fully establish whether there are true partner effects, and readers should not overgeneralize presented results, as a lack of significant results may not be sufficient evidence of null effects [50]. Additionally, our study was cross-sectional with a wide range of time since diagnosis; thus, causality cannot be determined, and longitudinal research is needed to identify mechanisms to determine when and how to intervene upon family systems to mitigate anxiety spillover processes. Our study also had proportionately more female than male caregivers, a characteristic common in pediatric oncology research [3, 10, 16, 29, 32]; future studies should aim to recruit more fathers to better understand why children with male caregivers may be reporting lower child overall health.

## Research and clinical implications

Despite limitations, our findings have important implications for practice and future research. First, our findings underscore supporting the broader family system during pediatric cancer, and particularly suggest benefits to mitigating caregiver anxiety [33]. Interventions are needed for caregivers and children to support mental and overall health during cancer treatment [59, 60], with findings suggesting tailoring support in particular to mothers and adolescents. Additional, fully-powered research is needed to better understand the mechanisms by which caregiver anxiety contributes to child overall health to further inform future interventions. Our results support soliciting multiple family members’ perspectives in pediatric oncology, as there appears to be both intrapersonal and interpersonal dynamics to explain patient overall health. Clinically, providing accessible and ample caregiver support is likely to have downstream effects on the child’s health and wellbeing.

## Conclusions

Our findings underscore supporting and soliciting reports from the family system within pediatric cancer and suggest the benefits of mitigating caregiver anxiety. Specifically, we found an individual’s own anxiety is a significant predictor of their perceptions of the child’s overall health and may also play a lesser role in the other dyad member’s assessment of the child’s health, though additional research is needed. Interventions are needed for caregivers and children to support their mental and overall health during cancer treatment, with potential added benefits from tailored support for mothers and adolescents.

## Data Availability

The datasets generated and analyzed during the current study are not publicly available but will be made available from the corresponding author on reasonable request.
